# The development, validation, and psychometric properties of the Japanese version of the Child Oral Health Impact Profile-Short Form 19 (COHIP-SF 19) for school-age children

**DOI:** 10.1186/s12955-020-01469-y

**Published:** 2020-07-11

**Authors:** Takao Minamidate, Naoto Haruyama, Ichiro Takahashi

**Affiliations:** 1 Section of Orthodontics and Dentofacial Orthopedics, Graduate School of Dental Science, Kyushu University, 3-1-1 Maidashi, Higashi-ku, Fukuoka-shi, Fukuoka, 812-8582 Japan; 2 Section of Orthodontics and Dentofacial Orthopedics, Faculty of Dental Science, Kyushu University, 3-1-1 Maidashi, Higashi-ku, Fukuoka-shi, Fukuoka, 812-8582 Japan

**Keywords:** Child oral health impact profile-short form 19, COHIP-SF 19, Oral health-related quality of life, Children, Questionnaire, Translation, Validation

## Abstract

**Background:**

This study was performed to develop and validate a Japanese version of Child Oral Health Impact Profile-Short Form (COHIP-SF) 19 and to assess its psychometric properties in Japanese school-age children.

**Methods:**

The original English COHIP-SF 19 was translated into Japanese (COHIP-SF 19 JP) using a standard forward and backward translation procedure. The psychometric properties of the COHIP-SF 19 JP were assessed in 379 public school students between 7 and 18 years of age in Fukuoka, Japan. Internal consistency (Cronbach’s alpha) and test-retest reliability (intraclass correlation coefficient, ICC) were the metrics used for evaluation of this questionnaire. The discriminant validly was examined using the Wilcoxon rank sum test to identify significant differences in COHIP-SF 19 JP scores according to the results of dental examinations. The convergent validity was examined using the Spearman correlations to determine the relationships between COHIP-SF 19 JP scores and the self-perceived oral health ratings. Confirmatory factor analyses (CFA) were performed to verify the factor structure of the questionnaire.

**Results:**

The COHIP-SF 19 JP revealed good internal consistency (Cronbach’s alpha, 0.77) and test-retest reliability (ICC, 0.81). Discriminant validity indicated that children with dental caries or malocclusion had significantly lower COHIP-SF 19 JP scores (*P* <  0.05); convergent validity indicated that the self-perceived oral health rating was significantly correlated with the COHIP-SF 19 JP total score and subscores (rs = 0.352–0.567, *P* <  0.0001), indicating that the questionnaire had a sufficient construct validity. CFA suggested that the modified four-factor model had better model fit indices than the original three-factor model.

**Conclusion:**

The collected data showed that the COHIP-SF 19 JP possesses sufficient psychometric properties for use in Japanese school-age children.

## Background

Oral health is an important component of overall health, as oral disorders can have a significant impact on physical, social, and emotional well-being [1]. For example, among oral disorders, dental caries (tooth decay) [2], missing teeth [3], malocclusion [4, 5] and craniofacial anomalies [6] can adversely affect quality of life (QoL). Therefore, oral health-related quality of life (OHRQoL) is becoming increasingly important in both population research and clinical practice [1].

A variety of questionnaires measuring OHRQoL have been developed for adults [7–9]. However, age-specific instruments are still required for better analysis of OHRQoL. As children and adolescents have different QoL-related issues compared to adults [10], various instruments for measurement of OHRQoL in pediatric populations have been developed over the past several decades, despite the difficulties associated with the development and validation of such instruments [11]. These include the Scale of Oral Health Outcomes for 5-year-old children [12], the Pediatric Oral Health-Related Quality of Life Measure [13] and the Child Oral Impacts on Daily Performances Index [14]. However, the most frequently used self-completed QoL scales for children are the Child Perceptions Questionnaire (CPQ) [15] and Child Oral Health Impact Profile (COHIP) [16].

The CPQ was the first validated questionnaire specifically designed to measure OHRQoL in children and adolescents [15]. The developers of the CPQ considered theories of child developmental psychology, and created different versions for specific age ranges, e.g. the CPQ_8–10_ for 8–10-year-old children and CPQ_11–14_ for adolescents [17, 18]. However, it is unclear whether the different versions are consistent, i.e. whether scores are comparable among them [19]. Therefore, care is required when conducting longitudinal research utilizing the CPQ [20].

The COHIP, which was originally developed to assess “oral–facial well-being” over a range of ages (8–15 years of age) [21] and ethnicities [22–24], is also a well-validated and comprehensive questionnaire for determining children’s OHRQoL. It contains 34 questions and five subscales (oral health, functional well-being, socio-emotional well-being, school environment, and self-image). The COHIP questions cover the oral and maxillofacial areas. An important characteristic of this scale is the inclusion of positive aspects of OHRQoL (e.g. confidence and attractiveness). Child Oral Health Impact Profile-Short Form (COHIP-SF) 19 is a shortened version of the scale, developed in 2012, containing 19 items and three subscales (oral health, functional well-being, and socio-emotional well-being); the psychometric properties of the original version are well-maintained [25]. The short form can be administered more quickly, which facilitates QoL assessment in clinical studies [20, 25]. COHIP-SF 19 has been translated into Arabic [26], German [19] and Chinese (Mandarin) [27]. As models of OHRQoL should take account of cultural factors [1], the COHIP-SF 19 is a good instrument for international comparisons of OHRQoL in child and adolescent populations. In addition, because its target age has been extended from 8–15 to 7–18 years of age [25], the COHIP-SF 19 is suitable for longitudinal research, such as QoL studies in patients with cleft palate who require long-term treatment commencing after birth and continuing into adulthood [20].

The present study was performed to develop and validate a Japanese version of the COHIP-SF 19 to assess its psychometric properties in Japanese school-age children. The development of different language versions of the COHIP is beneficial for future international comparisons of children’s OHRQoL as an outcome of clinical interventions in dental and craniofacial/maxillofacial regions.

## Methods

### Translation and pilot test of Japanese version of COHIP-SF 19 (COHIP-SF 19 JP)

This study protocol was reviewed and approved by the Kyushu University Institutional Review Board (IRB) for Clinical Research (#30–186). The original English version of the COHIP-SF 19 was obtained from the developer, then translated and adapted in accordance with standard guidelines [28]. The instrument was first independently translated into Japanese by two native Japanese speakers, both of whom were the dentists and fluent in Japanese and English. A native English speaker who was not familiar with the area of research discussed and revised the translation along with the two translators; a single consensus version was then compiled. This consensus version was pilot-tested on a sample of 20 young patients, 8–17 years of age, at the Department of Orthodontics, Kyushu University Hospital. Based on the feedback received from the participants, the members of the research team reviewed and modified the questionnaire for proper wording and layout. It was back-translated to English by a professional translator who was not familiar with the area of research. The back-translated English version was then evaluated by the developer and revised in accordance with her comments. Finally, the Japanese version of COHIP-SF 19 JP was compiled.

### Setting of variables

The COHIP-SF 19 JP questionnaire consists of 19 questions, which form three conceptual subscales: oral health (five items), functional well-being (four items), and socio-emotional well-being (10 items). Two of the 19 items were positively worded questions. Children were asked how often they had experienced oral impacts during the past 3 months; each question was answered using a five-point Likert scale, which ranged from “never” to “almost all the time.” Responses to the two positively worded questions were recorded as “never” = 0, “almost never” = 1, “sometimes” = 2, “fairly often” = 3, and “almost all the time” = 4. Scoring for the 17 negatively worded items was reversed. Therefore, higher scores reflected a more positive OHRQoL [25]. The overall score was calculated by summing the scores for all 19 items within a range of 0–76. In addition, there was one self-rated item concerning health/oral health, which was scored from excellent to poor.

### Data collection

Prior to data collection, we calculated the minimum sample size required for Cronbach’s coefficient alpha according to Bonett [29] with stringent parameters: minimum acceptable Cronbach’s alpha (H0), 0.6; expected Cronbach’s alpha (H1), 0.75; significance level (α), 0.05; two-tailed; power (1 − β), 90%; and number of items (k), 19. The minimum sample size needed was estimated as *n* = 102. For confirmatory factor analysis (CFA), we used ‘a rule of thumb’ for covariance structure analysis [30, 31], i.e. an ideal sample size-to-item ratio would be 20:1 (*n* = 380) and a less ideal ratio would be 10:1 (*n* = 190). We estimated a required sample size of 300 to allow both the reliability and structure analysis of the questionnaire.

All applied questionnaires were paper-based and self-administered. The cross-sectional study was conducted in Fukuoka, Japan, with a target population of students 7–18 years of age. Initially, four of 271 urban public schools were invited to participate in the study, and three schools (an elementary school, a junior high school, and a high school) agreed to participate in the study: all classes from grades 2 to 6 in the elementary school, which were equivalent to 8–12 years of age; all classes from each grade equivalent to 13–15 years of age in the junior high school; and two randomly selected classes from each grade equivalent to 16–18 years of age in the high school. The participating classes in the high school were randomly chosen by the school. The 20 patients who contributed to this pilot study did not participate in the survey.

The inclusion criteria [25] were as follows: age between 7 and 18 years, absence of cognitive impairment or other chronic illnesses, and no history of orthodontic treatment. The exclusion criteria [25] were as follows: age above or below the specified range, presence of cognitive impairments or other chronic illnesses, presence of severe oral pain and limited range of jaw movement, and current or prior orthodontic treatment. Prior to data analysis, participants who did not complete more than 75% of the questions in the COHIP-SF 19 JP were excluded. At the subscale level, if more than two thirds of the items were missing in a specific subscale, the sample was classified as missing. If fewer items were missing, the missing values were replaced with the mean score of available items. Of the 2043 children recruited for the study, the 520 responded, and the 379 fulfilled the inclusion/exclusion criteria and provided complete questionnaires that were usable for analysis. The reliability and validity of the COHIP-SF 19 JP were assessed based on these 379 participants. To assess the test-retest reliability, the same questionnaire was distributed to 210 participants who agreed to take the retest 3–4 weeks after the first survey. The “retest questionnaire” was administered only to those who provided written consent at the time of the initial test.

### Information regarding dental caries, gingivitis, plaque retention, and malocclusion

In addition to the questionnaire, we obtained general information regarding dental status for each participant from the annual dental examination conducted by the schools. Decayed-missing-filled teeth (df/DMF), gingivitis, plaque retention, and malocclusion were examined by fully trained school dentists, in accordance with the basic procedures of the Ministry of Education, Culture, Sports, Science and Technology of Japan [32]. The questionnaires were administered after the examination and collected within 1 month.

### Reliability and validity assessment

Internal consistency was assessed by calculating Cronbach’s alpha for the overall scale and for each subscale (oral health, functional well-being, and socio-emotional well-being). The magnitude of Cronbach’s alpha was judged in accordance with published guidelines [33]; a coefficient ≥ 0.7 was considered to indicate satisfactory internal consistency. In addition, Cronbach’s alpha for the instrument was computed for each item by sequentially removing the items from the instrument. Test-retest reliability was assessed using the intraclass correlation coefficient (ICC). Discriminant validity was also tested using four major indicators of dental status: dental caries, gingivitis, plaque retention, and malocclusion. Participants were divided into two groups according to the presence or absence of df/DMF, gingivitis, plaque retention, and malocclusion. The nonparametric Wilcoxon rank sum test was used to identify significant relationships between COHIP-SF 19 JP scores and the results of dental examinations. Convergent validity was assessed by measuring the coefficients of Spearman correlations between the self-perceived oral health rating score and scores from the COHIP-SF 19 JP. Self-perceived oral health ratings were surveyed using the independent question, “Overall, what is your oral health?”; the question was answered using a five-point Likert scale as “poor” = 0, “fair” = 1, “average” = 2, “good” = 3, and “excellent” = 4. These analyses were performed using JMP Pro 14 (SAS Institute Inc., Cary, NC, USA). In all analyses, *P* <  0.05 was taken to indicate statistical significance.

### Factor analysis

To evaluate factor loading of the subscales of the COHIP-SF 19 JP, confirmatory factor analysis (CFA) was conducted utilizing SPSS AMOS 26 (IBM Corp., Armonk, NY, USA).

The goodness of fit of the explored models was evaluated using several different model indices, including χ^2^/DF = chi*-*squared/degree of freedom, RMSEA = Root Mean Square Error of Approximation, GFI = Goodness of Fit Index, AGFI = Adjusted Goodness of Fit Index, CFI = Comparative Fit Index, AIC = Akaike Information Criterion. Values for acceptable fit were determined with reference to the literature [34], as follows: χ^2^/DF ≤ 3, RMSEA ≤0.08, 0.90 ≤ GFI, 0.85 ≤ AGFI, 0.95 ≤ CFI, AIC < AIC for comparison model.

## Results

### Descriptive statistics

The mean COHIP-SF 19 JP score was 60.7 (standard deviation [SD], ± 7.4) and the median was 62 (range, 32–76). Means, medians, ranges, and quartiles for all COHIP-SF 19 JP responses, with each subscale score, are shown in Table 1. The Shapiro–Wilk test showed that the distributions of overall COHIP-SF 19 JP scores and subscale scores were significantly different from a normal distribution (*P* <  0.001).

**Table 1:** Descriptive statistics for COHIP-SF 19 JP and subscale scores (*n* = 379)

Scale (possible range)	Mean (± SD)	Median (range)	1st quartile	3rd quartile
**Overall COHIP-SF 19 JP (0–76)**	60.7 (± 7.4)	62 (32–76)	56	66
**Oral health (0–20)**	15.6 (± 3.1)	16 (5–20)	13	18
**Functional well-being (0–16)**	14.2 (± 1.9)	15 (7–16)	13	16
**Socio-emotional well-being (0–40)**	30.9 (± 4.1)	32 (5–40)	29	40

The COHIP-SF 19 JP scores by sex and age were examined using the Wilcoxon rank sum test and the data are presented in Table 2. There were no significant differences in either the overall COHIP-SF 19 JP score or the subscale score for oral health according to sex or age. However, females had a significantly higher socio-emotional well-being subscale score than males (*P* = 0.02). There were significant differences in the subscale scores for functional well-being and socio-emotional well-being by age (school). Compared to the younger age group, the older age group had significantly higher functional well-being scores (*P* = 0.02) but significantly lower social-emotional well-being scores (*P* = 0.03).

**Table 2:** Descriptive analysis of COHIP-SF 19 JP scores by sex and age

	Sex			School		
	Male (*n* = 171)	Female (*n* = 208)		Elementary school 7–12 y (*n* = 190)	Middle school & High school 12–18 y (*n* = 189)	
	Mean (± SD)	Mean (± SD)	*P-*value	Mean (± SD)	Mean (± SD)	*P*-value
**Total COHIP-SF 19 JP**	60.21 (± 7.81)	61.09 (± 7.01)	0.27	61.11 (6.95)	60.27 (± 7.79)	0.44
**Oral health**	15.54 (± 3.25)	15.68 (± 3.07)	0.84	15.81 (2.87)	15.43 (± 3.40)	0.40
**Functional well-being**	14.22 (± 1.96)	14.10 (± 1.92)	0.50	13.94 (2.02)	14.37 (± 1.83)	0.02*
**Socio-emotional well-being**	30.44 (± 4.24)	31.30 (± 4.04)	0.02*	31.36 (4.08)	30.47 (± 4.18)	0.03*

*P <  0.05 by Wilcoxon rank sum test

### Reliability

Cronbach’s alpha of the total COHIP-SF 19 JP score was 0.77 (Table 3), indicating satisfactory internal consistency according to published guidelines [33]. Cronbach’s alphas for the subscales of oral health, functional well-being, and socio-emotional well-being were 0.57, 0.45, and 0.68, respectively (Table 3). The item-test correlation, item-rest correlation, and “alpha if item deleted” are indicated in Tables 3 and 4. The item-test correlation and item-rest correlation represent the correlations between an individual item and the total score, and between the item and the sum of the rest of the item scores, respectively. The “alpha if item deleted” represents the recalculated Cronbach’s alpha if each item was removed from the questionnaire. As shown in Table 4, the alpha increased slightly if the three items with the lowest item-rest correlations (Q7, Missed school for any reason; Q8, Been confident; Q15, Felt that you were attractive [good-looking]) were deleted.

**Table 3:** Internal reliability analysis of COHIP-SF 19 JP and each subscale (*n* = 379)

Scale (number of items)	Cronbach’s alpha	Item-test correlation	Item-rest correlation	Alpha if item deleted
**Total COHIP-SF 19 JP (19)**	0.77	0.08–0.68	0.04–0.60	0.74–0.79
**Oral health (5)**	0.57	0.36–0.56	0.23–0.45	0.75–0.77
**Functional well-being (4)**	0.45	0.31–0.48	0.24–0.37	0.76–0.77
**Socio-emotional well-being (10)**	0.68	0.08–0.68	0.04–0.60	0.74–0.79

**Table 4:** Item discrimination and reliability analysis of COHIP-SF 19 JP (*n* = 379)

Subscale	No.	Content	Item-test correlation	Item-rest correction	Alpha if item deleted
**Oral Health**	**Q1**	Had pain in your teeth/toothache	0.45	0.36	0.76
	**Q2**	Had crooked teeth or spaces between your teeth	0.55	0.43	0.76
	**Q3**	Had discolored teeth or spots on your teeth	0.54	0.42	0.76
	**Q4**	Had bad breath	0.56	0.46	0.75
	**Q5**	Had bleeding gums	0.36	0.23	0.77
**Functional well-being**	**Q9**	Had difficulty eating foods you would like to eat	0.44	0.35	0.76
	**Q13**	Had trouble sleeping	0.31	0.26	0.77
	**Q17**	Had difficultly saying certain words	0.34	0.24	0.77
	**Q18**	Had difficulty keeping your teeth clean	0.48	0.37	0.76
**Socio-emotional well-being**	**Q6**	Been unhappy or sad	0.62	0.55	0.75
	**Q7**	Missed school for any reason	0.08	0.04	0.78
	**Q8**	Been confident	0.24	0.09	0.79
	**Q10**	Felt worried or anxious	0.61	0.53	0.75
	**Q11**	Not wanted to speak / read out loud in class	0.41	0.30	0.77
	**Q12**	Avoided smiling or laughing with other children	0.58	0.51	0.76
	**Q14**	Been teased, bullied, or called names by other children	0.32	0.26	0.77
	**Q15**	Felt that you were attractive (good-looking)	0.23	0.10	0.78
	**Q16**	Felt that you look different	0.63	0.56	0.75
	**Q19**	Been worried about what other people think about your teeth, mouth, or face	0.68	0.60	0.74

In terms of test-retest reliability, the ICC was 0.81 for the overall COHIP-SF 19 JP score, which indicated good test-retest reliability. In addition, ICCs for the three subscales—oral health, functional well-being, and socio-emotional well-being—were 0.75, 0.67, and 0.76, respectively.

### Discriminant validity

Although the clinical dental indicators (df/DMF, gingivitis, plaque retention, and malocclusion) were recorded as continuous variables during the annual dental examination, the majority of variables had a value of 0 for all indicators. Therefore, we compared the COHIP-SF 19 JP total and subscale scores between groups with and without df/DMF, gingivitis, plaque retention, and malocclusion to analyze the discriminant validity (Table 5).

**Table 5:** Discriminant validity: COHIP-SF 19 JP scores based on the clinical dental indicators

	Sample No.	Total COHIP mean (± SD)	Oral health mean (± SD)	Functional well-being mean (± SD)	Socio-emotional well-being mean (± SD)
**df/DMF (−)**	272	61.01 (± 7.84)	15.81 (± 3.21)	14.34 (± 1.88)	30.86 (± 4.44)
**df/DMF (+)**	102	59.97 (± 6.07)	15.25 (± 2.84)	13.66 (± 2.05)	31.06 (± 3.36)
***P*-value**		0.03*	0.06	0.002*	0.49
**Gingivitis (−)**	355	60.77 (± 7.48)	15.69 (± 3.11)	14.13 (± 1.97)	30.96 (± 4.22)
**Gingivitis (+)**	19	59.79 (± 5.96)	15.16 (± 3.39)	14.53 (± 1.31)	30.11 (± 2.88)
***P*-value**		0.37	0.49	0.67	0.16
**Plaque retention (−)**	327	60.84 (± 7.53)	15.70 (± 3.15)	14.18 (± 1.96)	30.95 (± 4.24)
**Plaque retention (+)**	39	59.97 (± 6.02)	15.15 (± 3.01)	14.13 (± 1.58)	30.69 (± 3.36)
***P*-value**		0.25	0.24	0.40	0.48
**Malocclusion (−)**	281	61.33 (± 7.31)	15.80 (± 3.11)	14.26 (± 1.92)	31.27 (± 4.18)
**Malocclusion (+)**	85	58.82 (± 7.31)	15.12 (± 3.19)	13.92 (± 1.92)	29.79 (± 3.88)
***P*-value**		0.003*	0.06	0.09	0.001*

Note: Comparison of the total COHIP-SF 19 JP and scores of each subscale according to specific oral clinical outcomes. *P <  0.05

Children without df/DMF had significantly higher overall scores in total COHIP-SF 19 JP (*P* = 0.03) and functional well-being subscale (*P* = 0.002). Children without malocclusion also had significantly higher overall scores in total COHIP-SF 19 JP (*P* = 0.003) and the socio-emotional well-being subscale (*P* = 0.001). There were no significant relationships between plaque retention or gingivitis and COHIP-SF 19 JP scores.

### Convergent validity

The convergent validity of the COHIP-SF 19 JP is shown in Table 6. The average self-perceived oral health rating was 2.69 (± 0.99 SD). Correlations of total COHIP-SF 19 JP score or three subscale scores with the perceived oral health ratings were significant in all pairs (*P* <  0.0001), and the coefficients were positive (rs = 0.352–0.567).

**Table 6:** Convergent validity: Spearman correlations of the self-perceived oral health rating with the COHIP-SF 19 JP scores

	Perceived oral health	
	rs (*ρ*)	*P*-value
**Total COHIP-SF 19 JP**	0.567	< 0.0001*
**Oral health**	0.532	< 0.0001*
**Functional well-being**	0.352	< 0.0001*
**Socio-emotional well-being**	0.433	< 0.0001*

Note: Correlations of self-perceived oral health ratings with the total COHIP-SF 19 JP and each subscale score (*n* = 379). *P <  0.05

### Confirmatory factor analysis

**Fig. 1:**
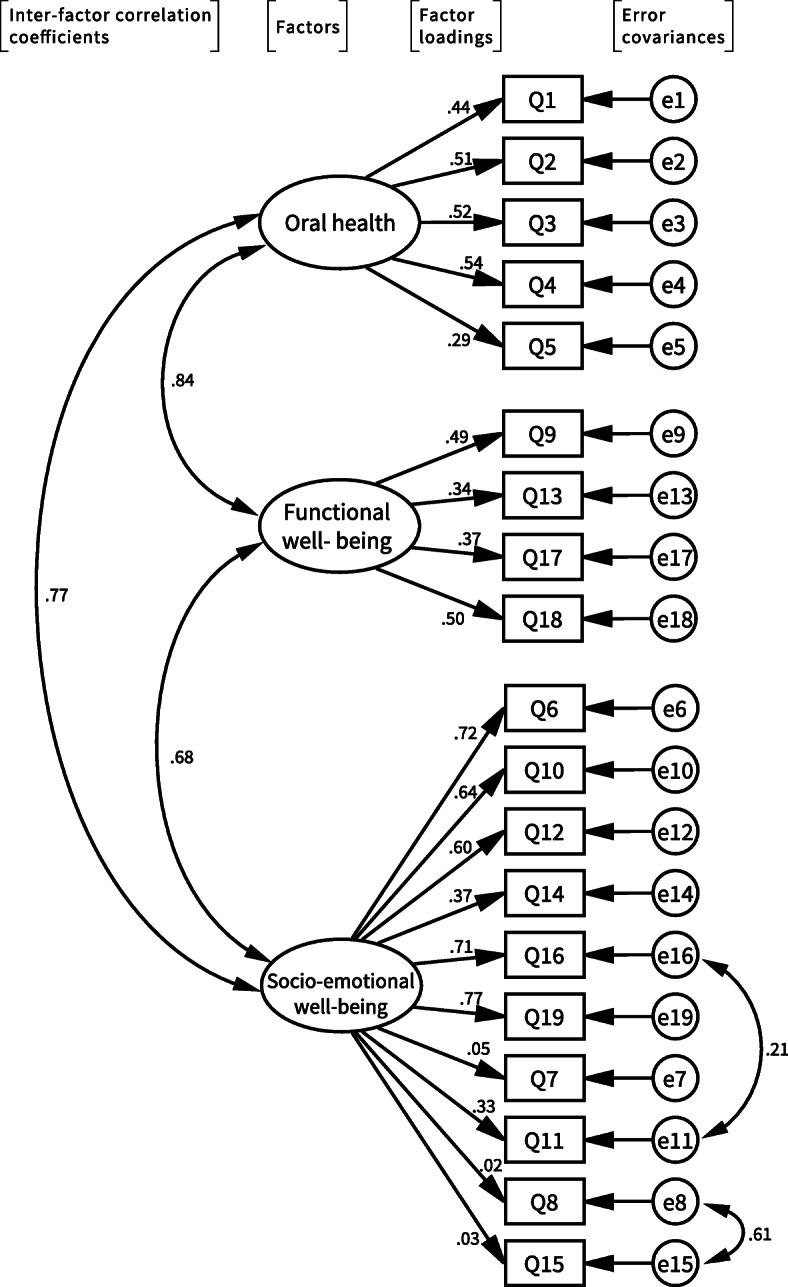
Three-factor model of COHIP-SF 19 JP by confirmatory factor analysis. This three-factor model maintained the same structure model as the original COHIP-SF 19. Inter-factor correlation coefficients showed correlations ranging from 0.68 to 0.84. Three items, Q7, Q8, and Q15, had small factor loadings < 0.1. Correlations of error covariances between two items were indicated only when correlations were > 0.2. Error covariances of Q8 and Q15 were strongly correlated

The fit values of the three-factor model, which is identical to the original COHIP-SF 19, satisfied the acceptable fit criterion (Table 7). Inter-factor correlation coefficients showed relatively higher correlations, ranging from 0.68 to 0.84 (Fig. 1). Three items (Q7, Q8, Q15) had small factor loadings of < 0.1. The strong correlation of the error covariances between Q8 and Q15 (0.61) suggested that the two items share some common characteristics, which are unique to them and are not well represented in the three-factor model.

**Table 7:** Comparison of measures of fit values of three-factor and four-factor models using CFA

Model	χ^2^	DF	*P*	χ^2^/DF	RMSEA	GFI	AGFI	CFI	AIC
**Three-factor**	353.88	147	< 0.001	2.41*	0.06*	0.91*	0.88*	0.86	439.88
**Four-factor**	346.50	145	< 0.001	2.39*	0.06*	0.91*	0.89*	0.86	436.50*

Note: *RMSEA* Root Mean Square Error of Approximation, *GFI* Goodness-of-Fit Index, *AGFI* Adjusted Goodness-of-Fit-Index, *CFI* Comparative Fit Index, *AIC* Akaike Information Criterion; Values for acceptable fit: χ^**2**^/DF ≤ 3, RMSEA ≤0.08, 0.90 ≤ GFI, 0.85 ≤ AGFI, 0.95 ≤ FI, AIC: smaller than AIC for comparison model [34]. * Satisfied acceptable fit criteria value

**Fig. 2:**
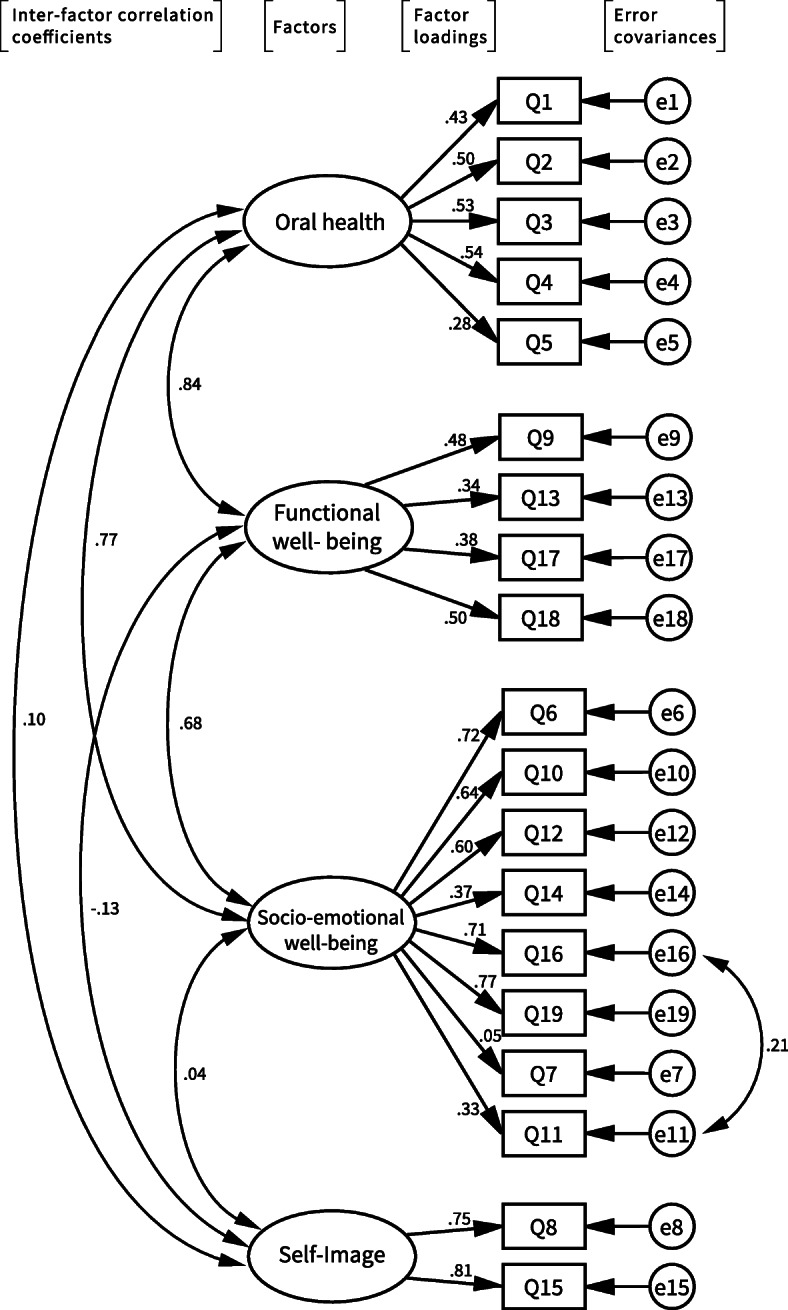
Four-factor model of the COHIP-SF 19 JP by confirmatory factor analysis. The four-factor model provided better factor loadings than the three-factor model. Inter-factor correlation coefficients remained high among the three existing subscales, but low correlations were observed with the new factor. Only one item, Q7, had small factor loading < 0.1. Correlations of error covariances between two items were indicated only when correlations were > 0.2

We explored a more reasonable model in which questions Q8 and Q15 were extracted as a new factor (Fig. 2). The factor loadings of the two positive questions, Q8 and Q15, were 0.75 and 0.81, respectively, indicating a better model for four potential factors. The two questions had belonged to an independent subscale known as “Self-image” in the original long-version, COHIP-34. This four-factor structure model showed slightly better fit indices in RMSEA = 0.06, GFI = 0.91, AGFI = 0.89, and AIC = 436.50, compared with the three-factor model [34] (Table 7). Inter-factor correlation coefficients among the three original factors were unaltered even in the four-factor model.

## Discussion

The development of a validated Japanese questionnaire is essential for the efficient and effective assessment of the OHRQoL of school-age children in Japan. This study was performed to adapt the original English COHIP-SF 19 to a Japanese cultural context and to test the psychometric properties of the COHIP-SF 19 JP in school-age children. In this study, the COHIP-SF 19 JP was developed in accordance with published guidelines [28], including the procedures, translation, back translation, conceptual equivalence confirmation by the original developer, and reliability and validity assessment with a population sample. Our results indicated that the COHIP-SF 19 JP possesses satisfactory psychometric properties for use in the targeted age group.

We have obtained 379 completed questionnaires for the assessments, which was sufficient for Cronbach’s alpha and covariance structure analyses, such as CFA. However, the response rate was relatively low. This may have been because the questionnaire was paper-based and answered at home rather than being completed at school; moreover, there was no compensation for participation, and each parent and child had to sign a consent form at home.

Interestingly, females had a significantly higher socio-emotional well-being subscore than males, which was opposite to the results obtained using the Dutch COHIP 34 [22] and Korean COHIP 34 [23]. A previous study using the Chinese COHIP-SF 19 found no sex differences in total or socio-emotional well-being subscores, but oral health and functional well-being subscores were higher in females than in males [27]. With regard to the differences that we noted according to age group, the increased functional well-being subscore in the older group may have been related to the fact that the younger (elementary school) group often had mixed dentition. In addition, the social-emotional well-being subscore was lower in the older age group, suggesting increased concern regarding appearance in Japanese adolescents. General health-related quality of life (HRQoL) has often been reported to be higher in adolescents than children, and females tend to show lower values than males [35, 36]. Future studies using COHIP-SF 19 JP should assess whether sex and age differences are due to specific characteristics of Japanese culture.

In this study, we investigated the internal consistency of COHIP-SF 19 JP reliability using Cronbach’s alpha coefficient. Cronbach’s alpha for the overall COHIP-SF 19 JP was 0.77, similar to Cronbach’s alpha in the original study (0.82–0.88) [25], the Chinese version (0.81) [27], and the German version (0.78–0.80) [19]; this finding indicated good internal consistency of the COHIP-SF 19 JP. Subscales showed relatively low Cronbach’s alpha values (0.45–0.68), although they tended to be similar to the Cronbach’s alpha values in the Chinese (0.59–0.74) and Arabic (0.57–0.67) versions. Notably, Cronbach’s alpha values for Oral health and Functional well-being subscales had lower scores than in previous studies, which may have been due to the small number of items included in these two subscales [37]. Three items (Q7, Missed school for any reason; Q8, Been confident; Q15, Felt that you were attractive [good-looking]) had relatively low item-rest correlations (< 0.2). These results were similar to the findings of the German version [19]; conversely, in the Chinese version [27], Cronbach’s alpha did not increase even if any of the items were deleted. Generally, items with inadequate psychometric properties may be either removed from the questionnaire or modified. However, these items were retained in our study, to allow comparison with international studies, as suggested by a study using the German version [19].

The test-retest reliability (ICC) for the overall scale of the COHIP-SF 19 JP was excellent and above the recommended threshold [38]. In addition, our ICC score for the overall scale was 0.82, which was higher than ICC scores observed in different language versions, such as the Arabic (0.76) and Chinese (0.77) versions [26, 27]; this finding suggested that the COHIP-SF 19 JP demonstrates sufficient stability over time.

Construct validity was assessed by examining discriminant and convergent validity [25]. The COHIP-SF 19 JP discriminant validity showed significant relationships with the statuses of dental caries and malocclusions. Children without dental caries reported a higher OHRQoL than children with dental caries; this finding was also observed with respect to malocclusion in the present study. Broder et al. [25] reported that US Latino children with dental caries in their permanent teeth had significantly lower scores in the overall COHIP-SF 19 and oral health subscale. Our data also showed that participants with malocclusion appeared to have lower COHIP-SF 19 JP scores than those without malocclusion, consistent with the findings of previous studies that used the Arabic and Chinese versions [26, 27]. Therefore, the COHIP-SF 19 JP exhibits sufficient discriminant validity for these oral clinical outcomes. However, gingivitis and plaque retention scores showed no significant associations with COHIP-SF 19 JP scores, which was presumably because the impacts of plaque and gingivitis on OHRQoL might have been insufficient for detection by the COHIP-SF 19 JP in our sample. The results regarding convergent validity of COHIP-SF 19 JP were satisfactory, as higher self-perceived oral health rating was associated with higher COHIP-SF 19 JP score, which was consistent with the findings of previous studies.

CFA is commonly used to examine the structure of instruments, such as OHRQoL [39–42]. With CFA, it is possible to specify precisely which items should load onto which factor. All relationships between factors and variables can be specified in advance in the model. Subsequently, the fit of the model to the data can be tested. An overall test statistic, along with a number of descriptive fit measures, may be obtained to evaluate the degree to which the model fits the data [43]. The main test statistic is the chi-square statistic. However, chi-square is an extremely sensitive statistical test, which is not interpretable in a standardized way and is not a practical test of model fit. Therefore, we used the chi-square/degree of freedom (χ^2^/DF) ratio, which is less affected by sample size [44]. In accordance with previous reports [34, 44], several different model indices were also used to evaluate the degree of fit of the data to the model, including RMSEA, GFI, AGFI, CFI, and AIC. RMSEA describes how closely the model fits the population, with lower values indicating better fit. GFI is a different type of measure where the model of interest is assigned a score between 0 and 1, with higher values indicating better fit. AGFI adjusts for the GFI model’s degrees of freedom relative to the number of observed variables, and therefore favors less complex models with fewer parameters. CFI is derived from comparison of the χ^2^/DF ratios between the null and alternative hypotheses. AIC adjusts the chi-square statistic for the number of parameters estimated, and can be used to compare competing models that do not need to be nested [34]. Our CFA demonstrated that the data collected with the three-factor model showed acceptable fit values, according to the previous report [34]; the three-factor model is the same structure as the original COHIP-SF 19. However, the CFA for the four-factor model provided better factor loadings and slightly better acceptable fit values, suggesting that the COHIP-SF 19 JP should include an additional factor. The two items, Q8 and Q15, composed the potential additional factor in our four-factor model. Exploratory factor analysis of the Arabic version of COHIP-SF 19 suggested that the four-factor model exhibited better fit, with the same two questions composed the additional factor [26]. These two items showed small factor loadings, strong correlation between error covariances in the three-factor model (Fig. 1), and low item-rest correlations (Table 4). Only these two items were actually positively worded questions, which originally belonged to a separate subscale, “Self-image,” with four other items in the full version of the COHIP-34. When developing the short version, only these two items remained, and were integrated into a single “Socio-emotional well-being” subscale. Although it appears inappropriate to include them in the Socio-emotional well-being subscale, we eventually chose to retain them for the purpose of international comparisons, as discussed above. However, care is needed when analyzing data from Q8 and Q15, as some characteristics of Japanese culture would have an influence. Indeed, modesty/humility is still one of the most important virtues in Japanese culture, so the positive questions regarding self-image may not reflect the QoL of Japanese children as well as those in other cultures.

Q7: Missed school for any reason because of your teeth, mouth, or face, also showed the smallest factor loading and the lowest item-rest correlation among all items. This was presumably due to differences in cultural background or the inclusion/exclusion criteria in our study. Because we excluded children who had current or prior orthodontic treatment, the majority of children requiring periodic visits to a dental clinic/hospital during the day had been omitted; this may have led to the high score with minimal variation. However, this question appears to be very important, especially when assessing the outcomes of patients with cleft lip and palate who frequently visit dental hospitals in Japan.

Use of the COHIP-SF 19 provides a number of opportunities for researchers and clinicians. Because of its short length, it places less burden on the patient, relative to the original 34-item version or other long instruments, such as the 37-item CPQ_11–14_ [15]. Another advantage of the COHIP is the wide age range, which allows longitudinal assessments that involve the COHIP in long-term prospective studies of treatment effects and prognoses. Moreover, the COHIP asks questions regarding the oral area and the maxillofacial region. For example, one question in the CPQ_8–10_ is “In the past 4 weeks, how often have you: Been concerned what other people think about your teeth or mouth?”; a similar question in the COHIP is “In the past 3 months, how often have you: Been worried about what other people think about your teeth, mouth, or face?”. Therefore, the COHIP may enable more effective evaluation when researchers and/or clinicians assess patients with diseases that affect the maxillofacial area (e.g. cleft lip and palate), which require a very long treatment period.

The International Consortium for Health Outcomes Measurement (ICHOM), has recently focused on the development of standardized datasets of subjective and objective outcomes and case-mix factors for use in clinical practice [45]. To measure the outcomes of patients with cleft lip and palate, the COHIP is recommended by the ICHOM for assessment of the oral area, along with CLEFT-Q [46]. Therefore, the development and use of the COHIP-SF 19 JP are likely to be especially beneficial for longitudinal studies or international comparisons of children’s OHRQoL.

This study had some limitations: the sampling was unbalanced and was conducted in a non-random manner. The difference between DMFT and malocclusion indices had a significant but modest impact on OHRQoL values. The sampling bias associated with the lower prevalence of oral diseases in urban schools could have led to the relatively low discriminant validity. In addition, the quality of clinical dental examination and the low response rate may also have affected the discriminant validity. As we did not use the results from the clinical examination specifically conducted for the purpose of this study, it was not possible to implement a calibration process for the examinations. In addition, the low response rate may have made the survey vulnerable to nonresponse bias. Evaluation of discriminant validity in additional pediatric populations is warranted. Further longitudinal and interventional studies may be required to better evaluate longitudinal validity and the sensitivity of the measurements. It should be noted that COHIP-SF 19 JP had better model fit indices when using the four-factor model.

## Conclusions

The Japanese version of the COHIP-SF 19 was validated in a representative community sample of 7–18-year-old Japanese schoolchildren. The COHIP-SF 19 JP was successfully developed in accordance with the standard procedure for cross-cultural adaptation of a self-reported instrument; it showed sufficient psychometric properties for use in Japanese school-age children.

## Data Availability

The datasets used and/or analyzed during the present study and the COHIP-SF 19 JP questionnaire are available from the corresponding author on reasonable request.

## Abbreviations

QoL: Quality of life
OHRQoL: Oral health-related quality of life
COHIP: Child Oral Health Impact Profile
COHIP-SF 19: Child Oral Health Impact Profile-Short Form 19
COHIP-SF 19 JP: Japanese version of Child Oral Health Impact Profile-Short Form 19
SD: Standard deviation
ICC: Intra-class correlation coefficient
CPQ: Child Perception Questionnaire
df: The number of decayed-filled teeth for deciduous or mixed dentition
DMF: The number of decay-missing-filled index for permanent dentition
CFA: Confirmatory factor analysis
χ^2^/DF: Chi-squared/degree of freedom
RMSEA: Root Mean Square Error of Approximation
GFI: Goodness of Fit Index
AGFI: Adjusted Goodness of Fit Index
CFI: Comparative Fit Index
AIC: Akaike Information Criterion
ICHOM: International Consortium for Health Outcomes Measurement
